# Revise the Belief in Loss Aversion

**DOI:** 10.3389/fpsyg.2019.02723

**Published:** 2019-12-03

**Authors:** Sumitava Mukherjee

**Affiliations:** Department of Humanities and Social Sciences, Indian Institute of Technology Delhi, New Delhi, India

**Keywords:** loss aversion, gains and losses, prospect theory, judgment, choice

## Do You Believe That Losses Loom Larger Than Gains?

Many might be promptly answering yes to such a question, especially those trained in psychology and applied behavioral sciences (like behavioral economics, medical decision-making, marketing, science communication, environmental action, or public policy). From the time Kahneman and Tversky (1979) proposed Prospect theory as an alternative to the dominant expected utility model in economics, the landscape of psychology (and recently, neuro/behavioral economics) has changed. Instead of the then-common idea of expected utility that explained the valuation of outcomes, Kahneman and Tversky suggested viewing possible outcomes as prospects by combining a value function and a probability function. The value function (with which we are concerned with here) was based on the loss aversion principle that states losses are weighted subjectively more than gains for the same objective magnitude, measured from a neutral reference point. This meant that the psychological value (or intensity) of losing (−500$) was much more than the value of gaining (+500$). The formal representation of the value function captures both risk aversion and loss aversion. The curvature of diminishing marginal utility explains risk aversion and an asymmetric slope at the origin codes differential subjective utility of gains vs. losses. Formally, the function is defined as a mapping from objective value (x) to subjective utility of the objective value *u*(*x*):

$$\begin{array}{l} If\;x\;\geq 0,\;u\left(x\right)=\;x^{\rho }\text{and }if\;x\;<0,\;u\left(x\right)=\;-\lambda \;{\left(-x\right)}^{\rho } \end{array}$$

where ρ is risk aversion constant and λ is loss aversion constant (commonly, λ > 1 signifying losses being psychologically more weighty than gains). When first introduced in 1980s, it was proposed as a reference-dependent theory of consumer choice. The applications from Prospect theory have been phenomenal and the theory is arguably one of the most influential ideas in the whole of social sciences (Camerer, 2005). There is no contention about Prospect Theory being a key insight that significantly influenced intellectual development in economics and psychology. Nevertheless, it is time to take a critical look (Gal and Rucker, 2018) in at least two folds: (i) what is loss aversion? and (ii) how confident are we about its empirical evidence?

## What is Loss Aversion?

The classic theorization as stated above specifies a well-defined mapping, which need not have any explicable process. It assumes no influence of context-sensitive processing just like some other static facts about (human) nature. Loss aversion is then a principle that can explain a myriad of phenomena like status quo bias, sunk costs and notably the oft discussed, endowment effect among others (Tversky and Kahneman, 1991; Kahneman, 2003, 2011). However, it has been used henceforth sometimes as a principle of human psychology while at other times as an explanation. For example, loss aversion was quoted as an explanation for endowment effect (Thaler, 1980; Kahneman et al., 1990) but at other times, endowment effect was quoted as a phenomenon that provided empirical evidence for loss aversion (Camerer, 2005). Thus, there is some amount of circularity such that loss aversion gets treated as a principle to predict phenomena and again, the same phenomena is used as empirical evidence for loss aversion. With regard to endowment effect, later studies have provided clarity on cognitive processes underlying endowment effect and showed possibility of multiple alternate explanations beyond loss aversion (Morewedge and Giblin, 2015). The first critical step is hence to decide how should we conceptualize loss aversion—is it a principle (beyond processes) or a phenomenon (with computational processes) or an explanation for other observable phenomena (with barely any non-trivial processes). Resolving this is critical for belief revision about loss aversion.

## Empirical Tests of Loss Aversion

Most previous studies have assumed loss aversion is true rendering it almost as a belief. For example, neuroeconomic studies often provide choices unto a point where the magnitude of gains is twice as much as losses (like +4 vs. −2$; Tom et al., 2007). This belief dates back to 1980s and has been held strongly until the present times. For example, “the value function is considerably steeper for losses than for gains” (Tversky and Kahneman, 1986, p. S255) and “The asymmetry is commonly thought to occur because people expect the pain of losing something to exceed the pleasure of gaining it” (McGraw et al., 2010, p. 1441). Although it was supposed to be a general hypothesis about “something,” most work was conduced only in the monetary domain. More importantly, loss aversion was stated as a principle, often beyond doubt and context. Whenever loss aversion did not show up, the “context” became “boundary conditions” (Novemsky and Kahneman, 2005), but loss aversion *per se* was not questioned empirically perhaps because a large number of published studies showed the framing effect of losses to be more affective than gains (for a review, see Camerer, 2005) although the file drawer problem could be a contributor too (Rosenthal, 1979).

Yet, a couple of studies did not continue the same belief and started to investigate the very existence of loss aversion treating it as a hypothesis subject to scientific scrutiny. One of the early studies that examined the predicted affect for gains and losses did not find evidence for loss aversion (Mellers et al., 1997). Further, even if people predicted losses would be more impactful than gains; when the outcomes were actually experienced, losses did not have as big an emotional impact as predicted (Kermer et al., 2006). These authors suggested that the purported asymmetrical impact of losses vs. gains was a property of affective forecasts and not of actual experiences. Harinck et al. (2007) and Mukherjee et al. (2017) further found even in affective forecasts when people made prospective judgments about how much intensity would a monetary outcome have; gains loomed equal to or larger than losses for low magnitudes while losses loomed larger for high magnitudes of money. McGraw et al. (2010) defended loss aversion in affective judgments by claiming that results which did not find loss aversion used a wrong measurement scale but Mukherjee et al. (2017) contended by showing that even by using the suggested way of measuring loss aversion as suggested by McGraw et al. (2010); loss aversion is not present all the time but is magnitude-dependent both for money and time (Ert and Erev, 2008; Mukherjee and Srinivasan, 2019; Yechiam, 2019). A range of studies examining phenomena related to loss aversion has not been able to confirm loss aversion thus raising questions about whether loss aversion is present at all and if so, when? We need to do more than plainly saying losses loom larger than gains (see Table 1 for studies which did not find losses always loom larger than gains).

**Table 1:** Some evidences against loss aversion.

**Phenomena that was linked to loss aversion**	**Challenged by**
Status quo bias	Gal, 2006
Endowment effect	Morewedge and Giblin, 2015; Yechiam et al., 2017
Risky bet premium	Ert and Erev, 2013; Yechiam and Hochman, 2013
Hedonic impact	Harinck et al., 2007; Mukherjee et al., 2017
Price elasticity	Mukherjee et al., 2017
Sunk cost effect	No neutral point and hence cannot be predicted

## Revisiting Loss Aversion

There seems to be at least three possible scenarios about loss aversion: (a) it is more contextual and nuanced than previously thought, (b) not observable most of the time, (c) superfluous as an explanation (Gal, 2006). If in the face of new empirical evidences, we do not assume that loss aversion is a principle (and hence is always true); then we should not conclude any evidences to the contrary as boundary conditions. It is indeed possible that empirical studies that found contradictions imply we need a theoretical update. Taking a soft stance would mean a position that claims loss aversion is more contextual and nuanced than previously thought. Accordingly, we can test new predictions in multiple domains like medical decisions, mobility behavior, health communication, etc., which will have important policy implications.

A way forward will be to try modeling loss aversion computationally that will break the black-box and take an information processing view so that we can unravel the cognitive processes underlying loss aversion. If it is a principle, then there is hardly anything to model. However, if it is a phenomenon, then we can attempt to detail the computations that lead to loss aversion. Studies have already linked loss aversion to attentional mechanisms (Yechiam and Hochman, 2013) and hence it does not seem likely that it is simply a bias but rather strategies involving information accumulation (Clay et al., 2017). We need more work to unravel the computational models that explain what are necessary and sufficient processes for loss aversion to occur (Lejarraga et al., 2019). Alongside, the neurological explorations have yielded a plethora of findings for about two decades (e.g., Gehring and Willoughby, 2002; Tom et al., 2007) and have given rise to neuroeconomics as a new field of enquiry. More recently, neuro-hormonal models of loss aversion are showing the intricate biological underpinnings of asymmetric valuation (Sokol-Hessner et al., 2009; Kandasamy et al., 2014; Sokol-Hessner and Rutledge, 2019). One way would be to let the computational process models use this new bio-behavioral data without assuming loss aversion as a constant (λ) and accordingly neither assume the slope nor the shape of the function but rather, let the data construct the affective value function. The more we are able to understand the computational details as these recent studies are doing, the more we will be closer to answer “what is loss aversion?” This is possible if we are convinced to update our long-held belief in loss aversion that has been deep-rooted for the past few decades.

The sociological belief in loss aversion is strong. I conducted a survey on intuitions about loss aversion (Mukherjee, 2019) on participants exposed to ideas in behavioral economics from different backgrounds (*n* = 71). It asked what did they believe in: (a) Gains loom larger than losses, (b) Losses loom larger than gains, or (c) Gains and losses have a similar psychological impact. These options were randomized and they had to choose one out of the three. 74.64% participants said they believed that losses loom larger than gains. Most disturbingly, the reasons cited for such a belief were responses like “from my experience” and “for most reasonable people, this should be the case.” The experience argument is difficult to test but if most people should believe losses loom larger than gains, then it goes against scientific scrutiny.

It seems that updating our belief in loss aversion will be an uphill task. However, doing so will advance the affective psychology of gains vs. losses and guide future developments and interventions. Multi-disciplinary investigations (behavioral, computational, and neurological) can help in breaking the belief-based approach to loss aversion, which stops treating it as a principle but more as a mechanism with clear processes (e.g., Clay et al., 2017; Yechiam et al., 2017; Lejarraga et al., 2019; Sokol-Hessner and Rutledge, 2019) to advance the questions in finer detail. Large-scale joint replication projects need to revisit the classic studies of Kahneman and Tversky while embracing heterogeneity (Owens, 2018; McShane et al., 2019) and then develop process-based computational models on those data to tackle both of the questions about loss aversion.

We need to start by not unanimously saying yes to the question “Do you believe losses loom larger than gains?”

## Author Contributions

The author confirms being the sole contributor of this work and has approved it for publication.

### Conflict of Interest

The author declares that the research was conducted in the absence of any commercial or financial relationships that could be construed as a potential conflict of interest.
